# Heteroaromatic *N*-Oxides in Asymmetric Catalysis: A Review

**DOI:** 10.3390/molecules25020330

**Published:** 2020-01-14

**Authors:** Zuzanna Wrzeszcz, Renata Siedlecka

**Affiliations:** Department of Organic Chemistry, Faculty of Chemistry, Wroclaw University of Science and Technology, Wyb. Wyspianskiego 27, 50-370 Wroclaw, Poland; zuzanna.wrzeszcz@pwr.edu.pl

**Keywords:** chiral heteroaromatic *N*-oxides, organocatalysis, asymmetric transformations stereoselectivity

## Abstract

An increasing interest in the synthesis and use of optically active pyridine *N*-oxides as chiral controllers for asymmetric reactions has been observed in the last few years. Chiral heteroaromatic *N*-oxides can work as powerful electron-pair donors, providing suitable electronic environments in the transition state formed within the reaction. The nucleophilicity of the oxygen atom in *N*-oxides, coupled with a high affinity of silicon to oxygen, represent ideal properties for the development of synthetic methodology based on nucleophilic activation of organosilicon reagents. The application of chiral *N*-oxides as efficient organocatalysts in allylation, propargylation, allenylation, and ring-opening of meso-epoxides, as well as chiral ligands for metal complexes catalyzing Michael addition or nitroaldol reaction, can also be found in the literature. This review deals with stereoselective applications of *N*-oxides, and how the differentiating properties are correlated with their structure. It contains more recent results, covering approximately the last ten years. All the reported examples have been divided into five classes, according to the chirality elements present in their basic molecular frameworks.

## 1. Introduction

Enantioselective organocatalysis is one of the more rapidly growing fields of research in modern organic chemistry. It provides the ability to effectively replace the enantioselective metal-containing catalysts with wholly organic molecules, while maintaining a high level of chemical efficiency and stereo control [1,2,3]. A key for success is the structural simplicity of the organocatalyst, which should be much more readily available than their organometallic equivalents. Among various Lewis base catalysts, those having the pyridine oxide moiety situated within a chiral environment, constitute a distinct class of highly active catalysts that are capable of high asymmetric induction, usually under mild reaction conditions. Since Nakajima’s report in 1998 [4] showing that axially chiral 2,2′- bipyridine *N*,*N’*-dioxides are effective as catalysts for the asymmetric allylation, the application of *N*-oxides has attracted considerable attention. This report outlines the most important and significant developments in catalytic uses of pyridine and bipyridine based *N*-oxides and their possible other applications, reported through the past decade. An observed increasing interest in chiral *N*-oxides can be explained by their advantages over metal-based catalysts, including their cost-effectiveness, low environmental harmfulness, and stability in the air [5,6,7]. In general, most of the heteroaromatic *N*-oxides have been obtained by oxidation. A simple and efficient procedure for the methyltrioxorhenium-mediated oxidation of pyridines has been developed by Sharpless [8] but it has several limitations. Procedures using *m*-chloroperoxybenzoic acid (*m*-CPBA) in mild conditions seem to be more common [9]. Alike preparation of various *N*-oxide derivatives makes it difficult to group them according to the structural features. It is easier to divide them by type of chirality they represent, defined as axial, central, helical and planar chirality. All these classes will be shown and briefly described in this work, along with the examples of catalytic applications in various enantioselective processes.

## 2. Chiral Heteroaromatic *N*-Oxides as Organocatalysts

**Scheme 1:**
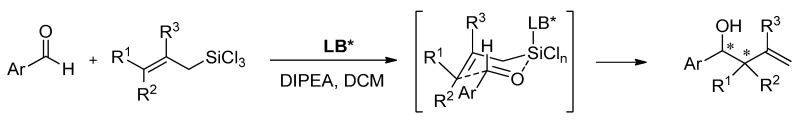
Lewis base (LB) catalyzed allylation of aldehydes with substituted allyltrichlorosilanes.

Properties of heteroaromatic *N*-oxides can be classified as strong Lewis bases because of N-O bond polarization [10]. They are able to activate the C-Si bond in halosilane compounds (Lewis acids), which makes them perfect mediators for allylation and crotylation of aldehydes with allyltrichlorosilanes, which are called Sakurai–Hosomi–Denmark-type reactions (see Scheme 1). This explains why allylation is the most popular testing reaction and is very well examined. It has also become a standard testing ground for new chiral Lewis basic organocatalysts [5,6,7,11]. The good selectivity is obtained when the catalyst allows the reaction to proceed via a closed cyclic chair-like transition state involving hypervalent silicates, as shown in Scheme 1. Additionally, the resulting homoallylic alcohols are considered the cornerstones of organic synthesis. Mostly, they are used in the synthesis of numerous natural products as building blocks but also in the synthesis of drug candidates and other functional molecules [11,12,13,14,15,16,17]. Apart from allylation there were also reported examples of using *N*-oxides in the catalytic ring-opening of meso-epoxides [18,19,20,21], propargylation [22], allenylation [23], aldol reaction [24,25] and reduction of ketoimines [26,27].

### 2.1. Axially Chiral N-Oxides and N,N′-Dioxides

**Figure 1:**
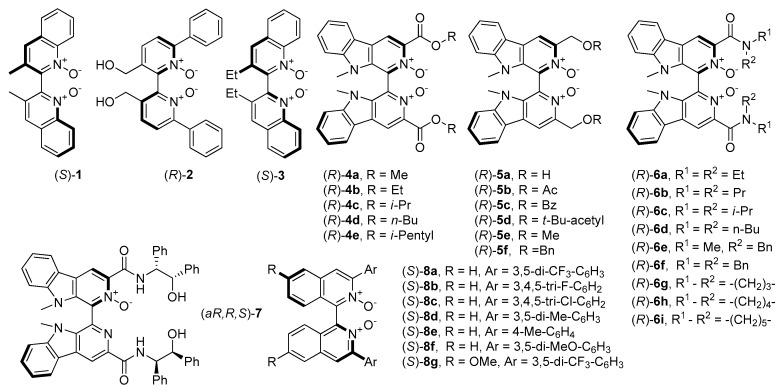
*N*-oxides possessing an axial element of chirality (*(aR*,*R*,*S)*-**7**, which possess also central chirality is placed here due to the similarity of the structure).

The first mention of *N*-oxides and their catalytic asymmetric applications were related to those, containing axial chirality and were developed by Nakajima et al. [4]. Initially, compounds were based on 4,4’-biquinoline and 2,2’-bipyridine *N*,*N′*-dioxide backbones (**1** and **2**, see Figure 1) which, apart from Nakajima’s work, were also investigated by Feng and coworkers [28]. A little later, Kotora et al. synthesized *N*-oxides with tetrahydroisoquinoline [29] and bis(tetrahydroisoquinoline) frameworks [30,31]. It is worth to highlight that organocatalysts **1** and **2** give, so far, one of the highest results (both in terms of yield and enantioselectivity) in the allylation of benzaldehyde with allyltrichlorosilane (85%, 88% ee (R) and 95%, 84% ee (S) respectively). Chang et al. received an analog of **1**, containing ethyl instead of methyl groups (**3**, see Figure 1) and describe their application as organocatalyst (10 mol%) in allylation of 4- metoxybenzaldehyde with allyltrichlorosilane. The chiral product was obtained with high enantiomeric purity (92% ee) with satisfactory yield 66% [32], which slightly exceeds the result obtained with the application of **1**. 

Reports concerning chiral biscarboline dioxide derivatives **4** and **5** were developed by Zhu et al. [33,34]. Executed screening of these compounds as benzaldehyde allylation catalysts showed, that the reaction yield for all types of catalysts was quantitative. In the case of use **4** also enantioselectivity was high (95%) while in the case of **5**, results were moderate (up to 82% ee). This implies that ester groups are involved in catalyzing this reaction. Catalysts **4a** and **4c** were chosen to test them in allylation of substituted benzaldehydes and aliphatic aldehydes. Independently from a substrate, using **4a** or **4c** (1 mol%) gave high enantioselectivity (91–97% ee or 53–90% ee respectively) and moderate to high yields (up to 90%). That makes these catalysts very versatile and applicable to different types of substrates, both these with electron-withdrawing and electron-donating groups. Furnished homoallylic alcohols (except for these ones from 2,6-dichlorobenzaldehyde and 3-phenylpropanal) had configuration *S*, so opposite to configuration of used catalysts (see Figure 1). 

Results of allylation using **4a** and **4c** are shown in Table 1. Catalysts **4a** and **4c** were also tested in allylation of benzaldehyde with different, substituted allyltrichlorosilanes, including crotylation (R^1^ = Me, R^2^ = R^3^ = H, Scheme 1). Generally, high ee values were achieved (up to 96%) but low to moderate yields (19–88%). The ratio of syn/anti products was the same as the ratio of *Z/E* isomers in the used substrate. Except for crotylation, in all other reactions slightly higher yields were obtained using catalyst **4c** but the ee values, obtained for both catalysts were similar. For catalyst **4c,** the impact of solvent was also tested. Enantioselectivity was satisfactory for all used solvents (85–95% ee) but the complete conversion was obtained only with solvents such as CH_2_Cl_2_ and CH_3_CN. For THF, toluene, EtOAc and Et_2_O conversion did not exceed 10%. It is also worth to mention that the syntheses of **4** and **5**, although multistep, were performed using simple transformations. Overall yield was about 40–50% for **4** and about 80% for **5**.

Zhu and coworkers have synthesized the chiral biscarboline *N*,*N’*-dioxide derivatives possessing secondary amide groups **6** (see Figure 1) [26,35]. Checking the effectiveness of **6a**–**b** and **6d**–**h** as catalysts in allylation of benzaldehyde with trichlorosilane (CH_2_Cl_2_, −80° C, 20 h), the authors focused on optimization the catalyst loading and, choosing the most effective catalyst, on examination of its application range [35]. The best results were observed for catalysts **6g** (84% ee) and **6h** (87% ee), which contained 4- and 5-membered cyclic amides, respectively. What is interesting, the highest enantioselectivity for **6h** has been obtained using 1 mol% of the catalyst. With other amounts: 0.1, 0.5, 5 or 10 mol%, the enantioselectivity was lower. The **6h** was applied in a series of reactions with different substrates. Generally, the resulted yield and enantioselectivity were good to excellent (12 examples, 85–97%, 67–96% ee). The influence of the position of substitution in benzaldehyde on the course of the reaction was tested. Mostly, *m*- and *p*-substituted benzaldehydes were compared. For electron-withdrawing groups, higher enantioselectivity was observed when the substituent was in position 3 (Table 1, entries 7, 10). The different situations took place for the electron-donating methoxy group. In this case, also *o*-substituted benzaldehyde was applied and, in all cases the yield was good (86–90%) but enantioselectivity increased in order: orto-, meta-, para-methoxybenzaldehyde (67, 83, 94% ee respectively, Table 1, entries 2, 6, 11). All obtained homoallylic alcohols had the configuration *R*, the same as the used catalyst. A comparison of the results of **6h** application with the described results of the use of structurally similar catalysts **4** and also with bisquinoline derivatives **8** are presented in Table 1. The catalyst **6h** was not very effective for aliphatic aldehydes (entry 32, Table 1). It was found that dichloromethane (DCM) was the suitable solvent, and as it was expected, the enantiomeric excess was strongly dependent on the reaction temperature.

Axially-chiral symmetrically substituted 2,2′-biquinoline *N*,*N’*-dioxide derivatives were developed by Takenaka and Peverati. Simple transformations allowed to receive the new Lewis base catalysts 8 (see Figure 1) with yields up to 99% [36]. Their catalytic utility was tested in allylation of 4-metoxybenzaldehyde with allyltrichlorosilane. Loading of 0.1 mol% was sufficient to get 83–96% conversion with enantioselectivity up to 96%. The best catalysts **8a**, **8b**, and **8d** were also used in the allylation of cinnamaldehyde. In this case, the most efficient was **8a** (having two bis 3,5-trifluoromethylphenyl substituents), which gave respective homoallylic alcohol with 84% yield and 92% ee. The obtained results were excellent, even at loading lowered to 0.05 mol%, for substituted aromatic, heteroaromatic and aliphatic aldehydes, but only if substrates were electron-rich aldehydes. The authors noticed that **8a** is less reactive and less selective for the halogen-substituted aldehydes. Considering the fact, that electron-rich Lewis base catalysts are usually more reactive in reaction with halosilane compounds, they decided to prepare compound **8g**, having two phenyl rings double substituted with CF_3_ groups, suitable for aldehydes with halogen groups [36]. Application of **8g** increased the yield, unfortunately with unchanged or only slightly increased enantioselectivity. The catalytic efficiency of **8a** and **8g**, compared with previously mentioned axial-chiral catalysts is listed in Table 1.

**Scheme 2:**
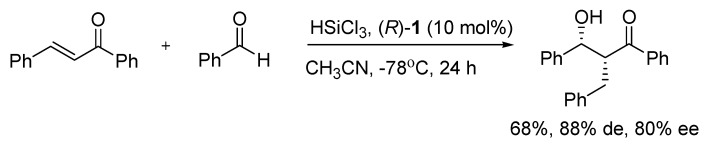
Reductive aldol reaction catalyzed with (*R*)-**1**.

Good activity and selectivity in the reductive aldol reaction of chalcone and benzaldehyde with trichlorosilane (Scheme 2) were obtained by employing bisquinoline *N*,*N’*-dioxide (*R*)-1 as a catalyst. It resulted in up to 80% ee of the product syn [25].

Zhu et al. have examined the catalysts **6b**–**e** and **6h**–**i** in the enantioselective hydrosilylation of ketoimines (Table 2) [26]. All tested *N*,*N’*-dioxides were effective; after 16 h (with 10 mol% of the catalyst) all the reactions were completed, but the enantioselectivity was moderate (6 examples, 42–77% ee). After optimization of the reaction conditions (1 mol% of catalyst in CH_2_Cl_2_ at 0 °C), catalyst **6i**, as the most effective, was used in the reduction of different ketoimines (11 examples). Obtained yield, in all cases, was very high (95–99%) however the enantioselectivity was up to 85% ee. Higher enantioselectivity was observed when R^1^ was an aromatic ring, furnished with an electron-donating group (reaction above Table 2). If phenyl in R^1^ had electron-withdrawing substituents, the asymmetric induction was lower. Except for three examples (Table 2, entries 7, 11, 13), obtained products had configuration *S*, opposite to used catalyst. 

The next approach presented by Zhu’s research group was the mono *N*-oxide **7**, with amide possessing additional stereogenic center [27], in order to improve the previously studied hydrosilylation of ketoimines. In fact, obtained results were excellent in many cases (14 examples, 94–98%, 75–96% ee), which makes the catalyst versatile and slightly more effective than 6i. A rough comparison of the catalytic efficiency of **6i** and **7** are given in Table 2. Application of **7** gave comparable yields and better enantioselectivity but requires higher catalyst loading.

### 2.2. N-Oxides Possessing Central Chirality

**Figure 2:**
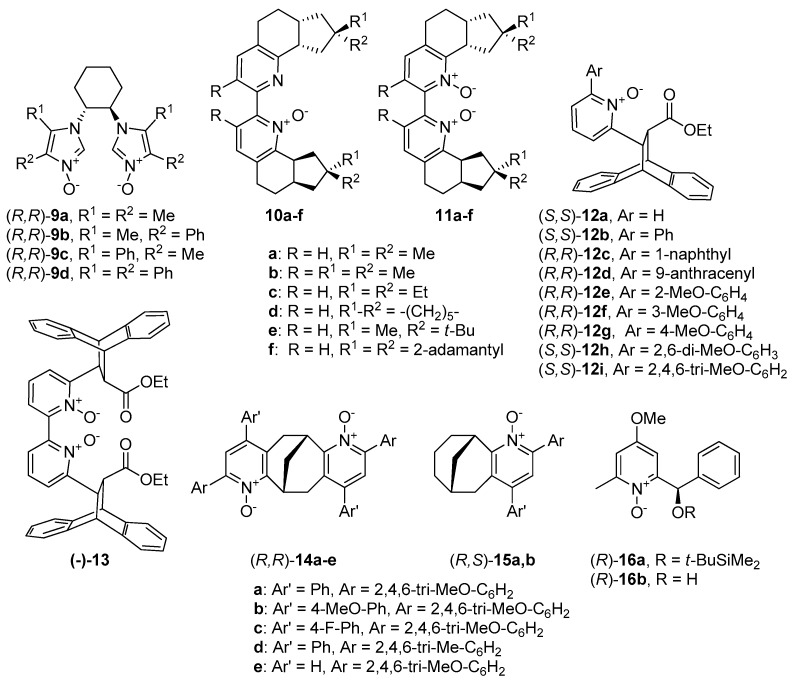
*N*-oxides possessing a central element of chirality.

Much more attention through the past decade has been given to compounds with central chirality. The report of Mlostoń and Jurczak from 2009 deserves to be mentioned [38]. Authors presented novel chiral *C2*-symmetric bisimidazole-*N*-oxides **9** (see Figure 2), derived from trans-1,2-diaminocyclohexane, thereby breaking the tendency of catalysts based on pyridine or bipyridine *N*-oxides. Screening of catalysts in the reaction of benzaldehyde with allyltrichlorosilane showed that the presence of phenyl group in the imidazole ring (R^2^ in **9b**) has a positive effect on the reaction efficiency (3 examples, 84–86%). Unfortunately, the introduction of two phenyl substituents (**9d**), only slightly increased enantioselectivity (from 43% ee to 53% ee). However, the presence of the second phenyl substituent in **9d** caused the inversion of the absolute configuration of obtained homoallyl alcohol. To improve the catalytic efficiency, different options of catalyst loading for (*R*,*R*)-**9d**, as well as reaction temperature was checked. The best result (90%, 64% ee) has been received for a reaction carried out at 0 °C with 10 mol% of (*R*,*R*)-**9d**. Also, estimation for a scope of aldehyde substrates was done. Higher enantioselectivity for *m*-substituted, than for *o*-substituted benzaldehydes was noticed. The highest asymmetric induction was observed for heteroaromatic aldehydes—furfural (76% ee) and thiophene-2-carboxyaldehyde (80% ee). 

Boyd presented the synthetic pathway to obtain bipyridine *N*-oxide derivatives **10** and corresponding *N*,*N*’-dioxides **11** [39], showed in Figure 2. It was found that the allylation reaction is slower when using the mono *N*-oxides, and thus, the reactions applying them were carried out at higher temperatures (0 °C or −40 °C, 24 h), compared with those using the corresponding *N*,*N’*- dioxides (−78 °C, 12 h). In that case, although the mono- and dioxides were applied to allylation of the same aldehydes with allyltrichlorosilane, it is difficult to compare the results unambiguously. The optimal enantioselectivity was observed in the allylation of 4-methoxybenzaldehyde as a substrate, using either *N*-oxides **10** (56–86% ee) or corresponding *N*,*N’*-dioxides 11 (59–80% ee), compared to allylation of benzaldehyde (24–35% *ee* and 14–26% ee). The highest enantioselectivity (86% ee) was observed in allylation of 4-methoxybenzaldehyde using 10b as a catalyst. Despite the clear difference in the enantioselectivity of these reactions, their yields did not differ much and were mostly in the range of 30–40% (except **10a**–60–72%). Better yields were observed when using **11** as the catalyst, and the compound 11a gave the highest induction (80% ee).

Conformationally rigid chiral backbone, with strong steric requirements, possessing an *N*-oxide unit was developed in 2012 by Ramanathan et al. [37]. Cycloaddition of anthracene and (E)-ethyl 3-(2-pyridyl)-propenoate, followed by the resolution of the enantiomers using l-(+)-tartaric acid, and then oxidation with *m*-chloroperoxybenzoic acid (*m*-CPBA) gave nine derivatives with usually good overall yields (up to 96%). All the chiral pyridine *N*-oxides **12** were evaluated in enantioselective allylation of 4-methoxybenzaldehyde (20 mol% of catalyst at −40 °C).

It was stated that an electron-rich catalyst causes greater enantioselectivity of the reaction using electron-rich aldehydes. The presence of alkyl or aryl groups at positions 2 and/or 6 of the pyridine ring reduces the nucleophilicity of the corresponding *N*-oxide. Therefore, the methoxy-substituted benzene rings (electron-rich aryl groups) have been attached at position 6 of the pyridine ring to afford the catalyst which can enhance both reactivity and selectivity. After optimizing the conditions, the reactions were carried out in a solvent mixture (CHCl_3_ and 1,1,2,2-tetrachloroethane in a 1:1 ratio—which enhances the yield and enantioselectivity), and the temperature was lowered from −40 °C to −78 °C. **12i** was determined as the most effective catalyst, which gave 84% yield and 87% ee in the case of 4-methoxybenzaldehyde and 81%, 94% ee for 3,4-dimethoxybenzaldehyde (see Table 1). In comparison to previously described catalysts **4a**, **c** and **8a**, **g**, for **12i** higher loading (20 mol%) was needed (results compared in Table 1). The advantage of 12i can be, that it can be used for unusual substrates. High enantioselectivity has been achieved in the allylation of heterocyclic and polycyclic aldehydes with **12i** (Table 1, entries 19, 21, 25). For others, results were rather moderate. Catalyst **12i** was also examined in the crotylation of 4-metoxybenaldehyde with crotyltrichlorosilane (*E/Z* = 82/12). The corresponding *anti/syn* alcohols were obtained in 81:19 ratio with 83% ee and 58% ee, respectively. 

Working with various enantiopure hydroxymethyl-substituted pyridine derivatives, Reissig and Eidamshaus received compounds **16** (see Figure 2), but using them as catalysts gave rather poor results [40]. Using 5 mol% of **16a** benzaldehyde was converted into homoallylic alcohol in 65% with 24% ee after 10 days at rt. Application of desilylated compound **16b** caused an increase in enantioselectivity to 47% ee but together with a simultaneous drastic decrease of yield to 12%. 

**Figure 3:**
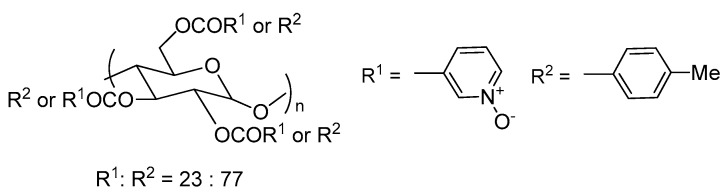
N-oxide derivative of amylose.

Interesting attempts to prepare polysaccharide (amylose and cellulose) derivatives, bearing pyridine *N*-oxide substituents, were made by the Ikai group [7]. A controlled number of 3- or 4-pyridine *N*-oxide groups have been attached to polysaccharide units by ester bonds. The great advantage of this type of compound is their non-toxicity to the environment. Unfortunately, the test applications in the reaction of asymmetric allyltrichlorosilane with benzaldehyde do not seem competitive with other described catalysts. The best results were received using amylose derivatives shown in Figure 3 (47–62%, 13–32% ee). It has been found that the amount and the position of *N*-oxide groups affected the reaction yield and enantioselectivity. Amylose not substituted with *N*-oxide groups do not show any catalytic activity. The increase of the number of *N*-oxide units in the catalyst from 19% to 23% caused improvement in both yield (from 47% to 62%) and enantioselectivity (from 13% to 32%). Further increase in the amount of pyridine *N*-oxide had a negative effect. Also, the location of the *N*-oxide groups was significant. 

Ramanathan group has tested the versatility of prepared derivatives as Lewis basic activators in the desymmetrization reaction of meso-epoxides with silicon tetrachloride [18]. Catalysts **12** applied in the reaction with *cis*-stilbene epoxide were not very selective (12a gave 32% ee, **12b**-5% ee and **12i**-42% ee). In contrast, the *C2*-symmetric bipyridine dioxide **13** with similarly conformationally rigid, chiral bicyclic skeleton gave 89% ee already in the initial trials. In subsequent experiments, the reaction conditions have been optimized in terms of the solvent used, the catalyst loading, reaction time and temperature, and the quantity of DIPEA used as a base. The best conditions for ring-opening of meso-epoxides were determined as 0.5 mol% of catalyst at −30 °C for 70 min, in CHCl_3,_ with 15 equivalents of DIPEA (see, reaction above Table 3). The results of the evaluation of (–)-**13** for the enantioselective desymmetrization of meso-epoxides of various structural classes are shown in Table 3. The best effectiveness was observed for *cis*-stilbene epoxide opening (94% yield, 93% ee, entry 1). One of the challenging substrates, cyclooctene oxide (Table 3, entry 7) successfully furnished the corresponding chlorohydrin in 84% yield with 69% ee, which is better result compare to obtained with bis(tetrahydroisoquinoline) *N*,*N*′-dioxides as catalysts (56% ee) [19].

**Scheme 3:**
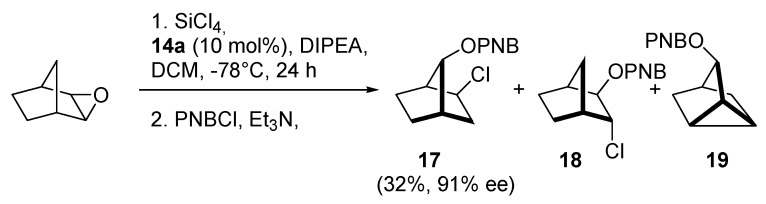
Catalytic ring-opening of norbornene oxide with SiCl_4_ [18].

Stončius and Neniškis presented a completely different approach. The Lewis bases were invented, in which the pyridine *N*-oxide unit has been attached to a chiral bicyclo [3.3.1] nonane backbone [18]. Authors designed the structures, that contain two 2,4-diaryl-substituted pyridine *N-oxide* moieties **14** and corresponding monofunctional congeners **15** (see Figure 2). Catalyst precursors were prepared in two steps: initial Michael addition was followed by the subsequent cyclization reactions to furnish corresponding bis(pyridines). Oxidation of them with *m*-CPBA produced expected *N*-oxides. Another synthetic strategy was taken for **14e**, which the authors briefly explained in the published work [9a]. The obtained catalysts were examined in the allylation of benzaldehyde with allyltrichlorosilane, but the results were mediocre, so they were tested also in the enantioselective ring-opening of meso-epoxides with silicon tetrachloride. Cyclohexene oxide ring-opening reaction was used as a model. The resulting yields were good, but enantioselectivity was far below expectations (7 examples, 72–85% yield, 3–47% ee). Better results obtained with the use of **14a** (72%, 32% ee), compared to **14d** (73%, 3% ee) suggest a beneficial effect of electron-rich 2,4,6-trimethoxyphenyl substituents on enantioselectivity. Catalysts **14a** and **14e** were also used in ring-opening of other epoxides, both cyclic and aromatic. Noteworthy is the result of opening the cyclopentene oxide with **14e** (85%, 88% ee)–the highest reported to date for the Lewis base-catalyzed desymmetrization of cyclic substrates. Any increase in ring size resulted in decreasing in selectivity (e.g., for cyclohexene oxide 77%, 47% ee). Interesting results have been obtained in the catalytic desymmetrization reaction of an epoxy norbornene derivative, known for that furnish syn, exo-chloroalcohol **17** as a major product, rather than vicinal chlorohydrin **18** (Scheme 3). For catalysts **14a**–**c** enantioselectivities of *p*-nitrobenzoate (PNB) **17** were excellent (90–96% ee) but yields were not higher than 50%. In other cases, the yield of reaction and asymmetric induction were low. Scale-up (for **14b**) or increase of catalyst loading (for **15b**) did not affect the yield or ee values but it was possible to isolate *p*-nitrobenzoate **19**. In turn, the reduction of catalyst loading (for **14b**) had a negative effect on reaction yield. Reduction from 10 mol% to 5 mol% caused a decrease in yield from 50% to 30%.

#### N-Oxides Having the Terpene Unit

**Figure 4:**
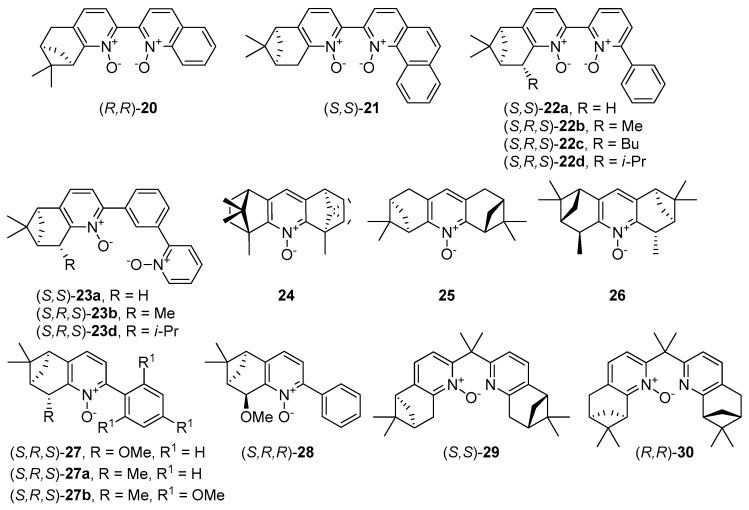
*N*-oxides possessing fragment from terpenes and alkaloids.

Naturally occurring and easily available terpenes and alkaloids are established chiral scaffolds for asymmetric organocatalysts. They were applied for the construction of chiral pyridine *N*-oxides, designed as a powerful Lewis-basic catalyst (Figure 4) already in the first years of the 21st century [41,42,43]. The latest reports about such catalysts come from 2008. Malkov and Kočovský [44] have prepared derivatives, lacking the *C2* symmetry and having the chirality ‘‘concentrated’’ on one side of the molecule, such as dioxide **20**-a quinoline analog with an isomeric terpene unit, the benzoquinoline analog **21** with similar chiral motif, and a series of bipyridine *N*,*N’*-dioxides with phenyl group **22** or pyridine *N*-oxide having (*α*-pyridyl-*N*-oxide)phenyl group **23**. All these compounds (in 10 mol% loading) were tested in standard benzaldehyde allylation reaction.

Chelucci et al. [45] presented pyridine *N*-oxide derivatives as a polycyclic structure with terpene fragments (**24**–**28**) and dipyridine monoxide with spacer (**29**,**30**), showed in Figure 4. Compounds **24**–**26** possess *C2*-symmetry. The catalytic activity and stereo-differentiating ability of the new compounds, tested in the allyltrichlorosilane addition to benzaldehyde, appeared to be poor. The yield did not exceed 65% and enantiomeric excesses were not higher than 48%. Application of compound **24** resulted in 65% yield, but isolated homoallylic alcohol was a racemate, while in the case of **25**, the obtained ee was 48%, but benzaldehyde was converted into alcohol in 31% yield. Epimers **27** and **28** behave as pseudo enantiomers and gave the opposite configuration of an allylic alcohol. Similar results of asymmetric induction have been achieved for both organocatalysts, **27** and its methyl analog **27a**. Due to the previously observed relationship in similar *C2*-symmetric structures, where enantioselectivity for mono *N*-oxide was higher than for *N*,*N’*-dioxide, compounds **29** and **30** were designed. Next to **29** and **30**, analogous *N*,*N’*-dioxides were also reported [46]. However, in these cases, the advantage of mono-oxides has not been observed. Compounds **24**–**26** and **29**–**30** were used also in the enantioselective opening of *cis*-stilbene. The yield achieved for all examined compounds was moderate to high (47–95%). Unfortunately, obtained halohydrin in most experiments was a racemate. Only for catalyst **25** asymmetric induction occurred (37% ee).

**Scheme 4:**
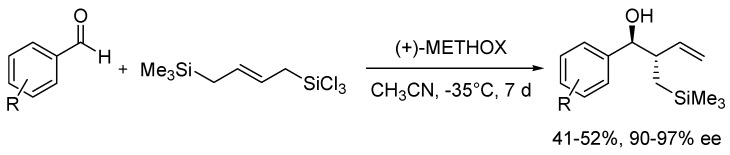
Allylation of aldehydes with allyldisilane catalyzed by (+)-METHOX.

**Figure 5:**
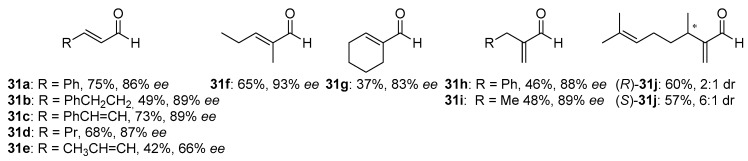
The scope of the reaction employing (+)-METHOX for *α*,*β*-unsaturated aldehydes.

METHOX (**27b**), developed and successfully used in allylation of aromatic aldehydes with allyltrichlorosilane a few years earlier [47], in 2011 was tested in similar reaction but using allyldisilane (Scheme 4) [48]. Catalytic reactions of allyldisilane with aldehydes proceeded at very low rates and required a week or more to reach completion (10–15 mol% of (+)-**27b**, at −35 °C, in CH_3_CN). Nevertheless, METHOX exhibited excellent diastereo- and enantioselectivities for benzaldehyde and its derivatives (up to 97% ee). Further, this methodology was extended to the group of *α*,*β*-unsaturated aldehydes [48,49]. Different *α*,*β*-unsaturated aldehydes **31**, incorporating aromatic, aliphatic substituents and cyclic ones were examined, and the results are shown in Figure 5. All of the aldehydes, with or without the α-branching, were found to react with good conversion within 3–5 days, providing high enantioselectivity, up to 93% ee. However, slightly better effectiveness was observed for **31a**–**c** and **31f**. In other cases, obtained yields varied between 37–60% and measured asymmetric induction from 66% ee to 89% ee. Received products were levorotary (except for (*S*)-**31j** use) when (+)-METHOX was employed. Aldehyde **31d**, crotylated under the same conditions, gave rise to the expected anti-diastereoisomer as practically the only product, with the highest enantiomeric excess (96% ee). METHOX has been found to exhibit a high tolerance to aldehyde electronics, and the reactions require low catalyst loading (even 5 mol%).

**Figure 6:**
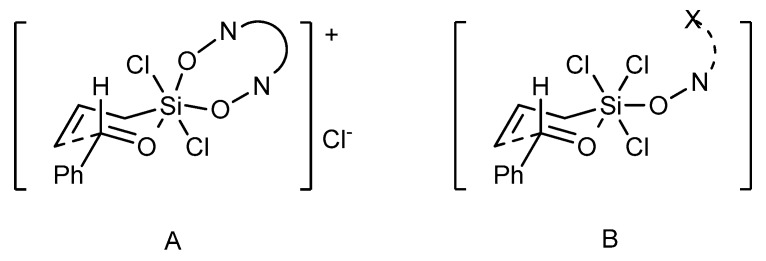
Envisioned transition states in the *N*-oxide catalyzed allylation of aldehydes [44].

The observed difference in the reactivity and selectivity of dioxide and monoxide catalysts suggests that these two catalyst types can operate *via* different mechanisms [44]. In the case of bidentate *N*,*N’*-dioxides, cationic transition state **A** can be envisioned (see Figure 6). On this basis, a significant impact on the observed enantioselectivity of both, the unsymmetrical catalyst substitution and the huge effect of axial chirality of the catalyst can be explained (determining the absolute configuration of the product). Alternatively, the reaction proceeds via an associative pathway, involving the neutral octahedral silicon complex **B**. Transition state **B** provides high enantiocontrol in the allylation of aromatic aldehydes, however, it can be sensitive to the electronic effects of substituents in a substrate and any variation in the catalyst structure proximal to the coordinating center.

### 2.3. N-Oxides with Central and Axial Chirality

**Figure 7:**
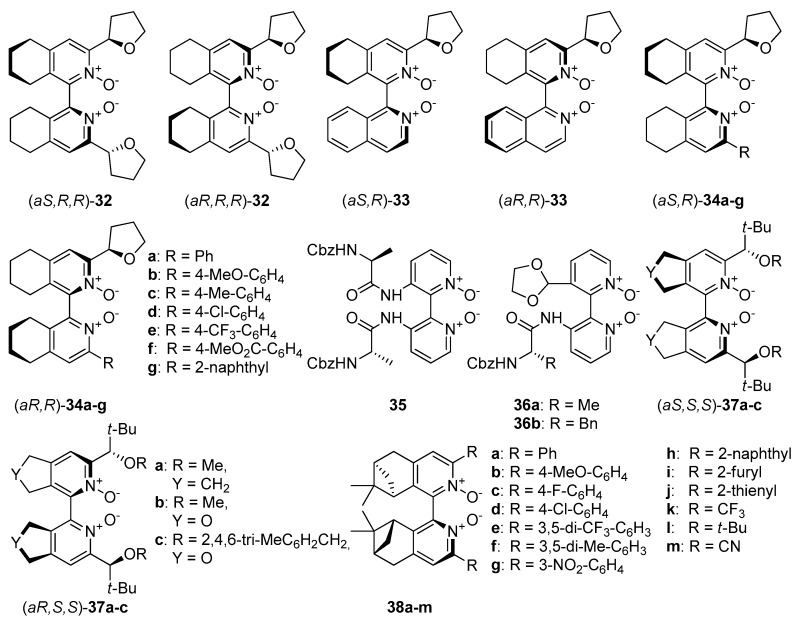
*N*-Oxides possessing two types of chirality—central and axial.

An interesting direction in the exploration of catalytically useful *N*-oxides is the combination of two types of chirality. Researches mainly concentrate on symmetrically or unsymmetrically substituted chiral bis(tetrahydroisoquinoline) *N*,*N’*-dioxides (Figure 7), which with their structure resemble compound **8**. In 2008 Kotora et al. synthesized symmetrically substituted by (*R*)-tetrahydrofuran-2-yl dioxides **32** (see Figure 7), which were tested in the allylation of aromatic [50] as well as aliphatic aldehydes [30]. Catalysts synthesis was based on cyclotrimerization of tetrayne with (*R*)-tetrahydrofuran-2-carbonitrile, followed by oxidation of received bipyridines by *m*-CPBA. At the end separation of a resulted mixture of diastereoisomers was necessary. A simple column chromatography on alumina, gave isolated yields 48% for (*aR*,*R*,*R*)-32 and 28% for (*aS*,*R*,*R*)-**32**. The configuration was assigned by X-ray crystallographic analysis. In allylation of benzaldehyde, 4-trifluoromethylbenzaldehyde or 4-methoxybenzaldehyde performed in MeCN, at −40 °C for 1 h, with 1 mol% catalyst loading, both diastereomeric catalysts were effective [50]. Aldehydes were converted into corresponding homoallylic alcohols almost quantitatively (Table 4, entries 1, 4, 10). Only for allylation of 4-trifluoromethylbenzaldehyde using (*aR*,*R*,*R*)-**32** the yield was slightly lower (82%, entry 10). Applying the same catalyst, the enantioselectivity was lower for aldehyde with an electron-withdrawing group (15% ee in comparison to 48% ee for benzaldehyde, Table 4, entry 10) and higher for aldehyde substituted with an electron-donating group (60% ee, Table 4, entry 4). When (*aS*,*R*,*R*)-**32** was used decrease of asymmetric induction for 4-trifluoromethylbenzaldehyde was observed again (Table 4, entry 10), but for 4-methoxybenzaldehyde obtained alcohol was a racemate (Table 4, entry 4).

In addition, a strong solvent effect on stereoselectivity has been observed. For (*aR*,*R*,*R*)-**32** used in chlorobenzene no reaction was observed, but using (*aS*,*R*,*R*)-**32** the enantioselectivity was significantly higher (Table 4, entries 2, 5, 11), for all three substrates. For benzaldehyde yield did not change, while for substituted substrates it decreases by half. Inversion of product configuration was observed in dependence of solvent used–*R* in MeCN into *S* in PhCl. Kotora also mentioned in the same publication about isoquinolyl-tetrahydroisoquinoline **33** unsymmetrically substituted with tetrahydrofuran-2-yl group. The catalysts themselves were obtained in low yields (4–17%), and their use for allylation of benzaldehyde gave rather mediocre results (86% with 49% ee using (*R*,*R*)-**33** and 84% with 48% ee using (*S*,*R*)-**33**). However, configurations of received homoallylic alcohols (*S* with (*R*,*R*)-**33** and *R* with (*S*,*R*)-**33**) indicates that configuration of the product is controlled by the axial chirality of the catalyst (the opposite is obtained). The catalytic activity of **32** was also studied in the allylation of aliphatic aldehydes. Different solvents e.g., acetonitrile, dichloromethane, chloroform, and acetone have been tested in allylation of cyclohexanecarboxaldehyde as the model substrate and there is no doubt that the solvent effect controls the reaction mechanism. Using (*aR*,*R*,*R*)-**32** enantioselectivity was low, regardless of the solvent used (10–19% ee, Table 5, entries 1–4), but the yield proved to be highly dependent on the solvent. The highest yield was obtained when MeCN was applied (85%, Table 5, entry 1). For the remaining solvents, the yields were similar and amounted to 34–40%. Application of (*aS*,*R*,*R*)-**32** resulted in an increase of both the yield (52–79%, Table 5, entries 5–8) and the reaction enantioselectivity (39–68% ee). Configuration of obtained alcohol was inverse to those obtained with (*aR*,*R*,*R*)-**32**, which confirms prior observation about the decisive influence of the axial chirality of the catalyst. (*aS*,*R*,*R*)-**32** employed toward other aliphatic substrates gave generally high yield (79–91%, Table 5, entries 9–12) except for pivaloyl aldehyde (10%, Table 5, entry 13) but enantioselectivity was rather moderate (22–68% ee). For cyclic substrates, products have configuration *R* (Table 5, entries 11–12), while straight-chain aldehydes resulted in alcohols with configuration *S* (Table 5, entries 9, 10, 13).

The unsymmetrically substituted bis(tetrahydroisoquinoline) *N*,*N’*-dioxides **34a** were also synthesized and employed in catalytic allylation of aromatic aldehydes (see Table 4) [31]. Again, the huge influence of the solvent on the stereochemical result of the reaction was observed. The catalyst (1 mol% loading) with *S*-axial chirality gave *R*-product in MeCN (Table 4, entry 1) and *S*-product in PhCl and THF (Table 4, entries 2,3). Also, in MeCN the enantioselectivity was about two times lower than in PhCl and THF, for which results were similar. Therefore, reactions with other substrates were carried in THF. Generally, for both isomers, **34a** results were very good, but (*aS*,*R*)-**34a** worked better as a catalyst for highly enantioselective allylation of benzaldehydes, bearing electron-withdrawing as well as electron-donating groups. Slightly better yield can be observed in the case of *p*-substituted benzaldehydes than for *m*-substituted (Table 4, entries 5–9, 11–14). A drastic decrease in the yield and asymmetric induction was observed for *o*-chlorobenzaldehyde (Table 4, entry 15). Results depicted in Table 4 clearly show, that unsymmetrically substituted bis(tetrahydroisoquinoline) *N*,*N’*-dioxides **34a** exhibit higher catalytic activity, than symmetrically substituted derivatives **32.** Yields of obtained products are comparable, but much better enantioselectivity gives the use of **34** (both epimers). The enantioselectivity was highly solvent-dependent. It seems, that THF enables the reaction mechanism to proceed through the sterically more crowded neutral six-coordinate silicon species, leading to higher enantioselectivity [30]. The scope of application of **34a** for aliphatic aldehydes was tested in the reactions with *n*-octanal or cyclohexylcarbaldehyde (Table 5, entries 14–17) [31]. The reaction in both cases was characterized by good to excellent yields (88–95%) and moderate enantioselectivity (38–67% ee).

**Scheme 5:**
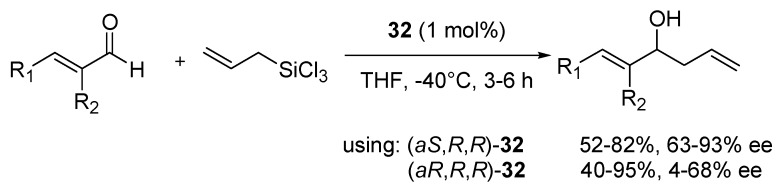
Allylation of *α*,*β*-unsaturated aldehydes with allyltrichlorosilane catalyzed by **32** [51].

Higher results were detected for (*aR*,*R*)-**34a**. (*aR*,*R*)-**34a** was also applied in allylation of benzaldehyde with *E*- crotyltrichlorosilane (6:1 *E/Z* mixture in PhCl, for 24 h, at −40 °C with 1 mol% of catalyst), giving 2.4/1 anti/syn diastereoisomer mixture, with good yield 82% and satisfactory enantioselectivity 91% and 87% ee, respectively. The scope of application in the asymmetric allylation reaction of both, symmetric catalysts **32** was studied [51]. Under optimized conditions (1 mol% of the catalyst in THF at −40 °C) a number of different, aromatic and aliphatic *α*,*β*-unsaturated aldehydes were checked, testing both epimers of **32** (Scheme 5). Better enantioselectivity was achieved using (*aS*,*R*,*R*) diastereomer but slightly higher yields were obtained for (*aR*,*R*,*R*) diastereomer–respectively, 52–82% yield, 63–96% ee and 40–95% yield, 4–68% ee. The best enantioselectivity was achieved using (*aS*,*R*,*R*)-**32** with substrates having both groups: R^1^ (as phenyl) and R^2^ (as methyl group or chlorine atom)-96% ee in both cases. By using (*aR*,*R*,*R*)-**32** it was found that, that higher enantioselectivity (62–68% ee) was obtained when α-substitution (R^2^) was present independently of whether R^1^ was aromatic or aliphatic. Better yields for (*aR*,*R*,*R*)-**32** (82–97%) were observed when R^1^ was phenyl substituted with an electron-withdrawing group or unsubstituted phenyl group, but then the presence of electron-donating group as R^2^ was necessary. For both catalysts employed, the configuration of obtained aliphatic alcohols was *R*, whereas it was opposite for products having an aromatic substituent. In most applications, both catalysts appear to be similarly effective to the METHOX described earlier.

**Scheme 6:**
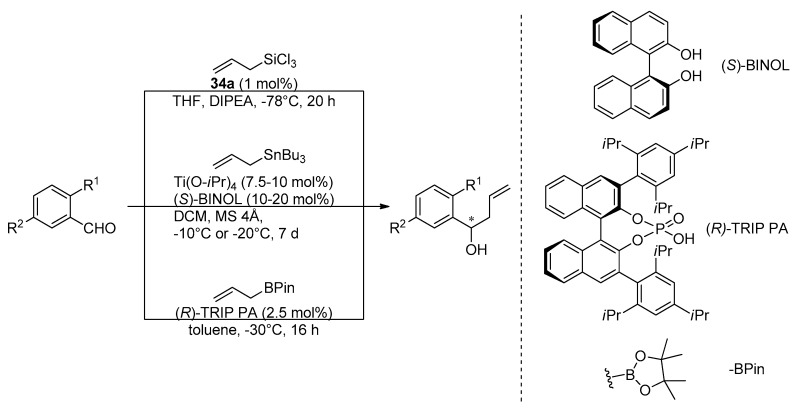
Enantioselective allylations of *o*-substituted benzaldehydes carried out in comparative studies [52].

Interesting comparative studies have been done by Kotora’s research group for different enantioselective allylation procedures and different catalysts applied *N*-oxides **34a**-Lewis basic catalyst were compared with (*S*)-BINOL-Lewis acid catalyst (Keck protocol) and (*R*)-TRIP PA-Brønsted acid catalyst in enantioselective allylation of *o*-substituted benzaldehydes (Scheme 6) [52].

**Scheme 7:**
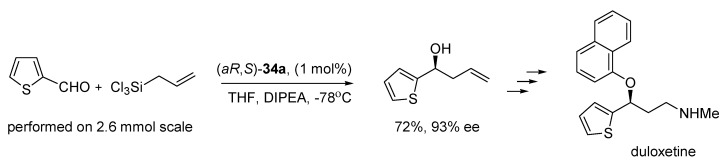
Allylation of 2-thiophenecarboxaldehyde using (*aR*,*S*)-**34a** in total synthesis of duloxetine [12].

The reactions performed needed different catalyst loadings and different reaction times. Despite this, an attempt to compare the results was made. Chiral catalytic systems have rarely been used for allylation of *o*-substituted benzaldehydes, so the effect of substitution in the ortho position of the aromatic aldehydes was tested using the three procedures shown in Scheme 6. Definitely, the best yields were obtained using phosphorous catalyst (*R*)-TRIP PA (93–99% in comparison to results up to 70% for **34a** and up to 80% for (*S*)-BINOL), but achieving high enantioselectivity was problematic in all cases. In some cases the use of *N*,*N’*-dioxide catalysts gives better optical purity, in others, Keck or Brønsted acid allylation seems to be more effective. Also, none of the catalysts were versatile. Among tested *N*,*N’*-dioxides, again better yields gave (*aR*,*R*)-**34a** and higher asymmetric induction was observed using (*aR*,*S*)-**34a**. The best results were obtained for *o*- fluorobenzaldehyde (48%, 82% ee for (*aR*,*R*)-dioxide and 34%, 66% ee for (*aR*,*S*)-dioxide), *o*-vinylbenzaldehyde (40%, 72% ee and 40%, 76% ee, written in the same order) and *m*- methoxybenzaldehyde (60%, 85% ee and 60%, 88% ee), which are rather moderate. The effect of the solvent was examined in allylation of 2-iodo-5-methoxybenzaldehyde. For (*R*,*R*)-epimer reaction proceeded better in dichloromethane than THF—70%, 41% ee in comparison to 5%, 56% ee and reversely, in case (*R*,*S*)-enantiomer-40%, 4% ee versus 53%, 80% ee, whereas in toluene reaction did not proceed at all, in both cases. When the reaction was performed in THF, the configuration of all products was the same as the axial configuration of the catalyst. Inversion of product configuration was observed when dichloromethane was applied. *N*-oxide catalysts were successfully applied to the synthesis of a few natural products [12,13,14,15,16,17]. For example, duloxetine, which is used in the treatment of major depression, was synthesized involving the allylation of 2-thiophenecarboxaldehyde with (*aR*,*S*)-**34a** as catalyst (see Scheme 7) [12]. 

**Scheme 8:**
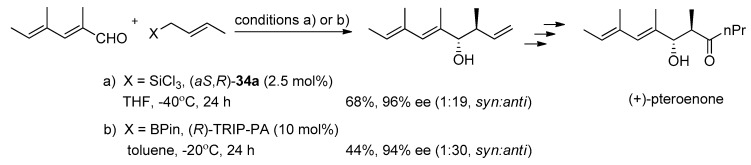
Comparison of the crotylation involving *N*,*N’*-dioxide **34a** and TRIP PA used as a catalyst in the total synthesis of (+)-pteroenone [15].

The advantage of the N,N’-dioxide catalysts is in their rather low loading, (only 1 mol%) being sufficient to bring about the desired allylation. In the case of phosphorous catalyst TRIP PA, the level of asymmetric induction depends on the catalyst loading, optimally it is 10 mol%, which is rather high (Scheme 8) [15].

Malkov and Kočovský [48] have compared the effectiveness of catalyst **34a** and METHOX in the reaction of different aldehydes with allyldisilane. Generally, obtained results were high and similar for both catalysts, however, reactions applying METHOX needed higher loading (15–20 mol% versus 5–10 mol%) and much longer time (7 days versus 12 h) to be completed. Optimal solvent for use of **34a** as catalyst was THF, while CH_3_CN for METHOX (temp. −35 °C for both). At the enantioselectivity of a similar order, the yields obtained were slightly higher for **34a** (for both isomers 71–83% yield and 73–98% ee). In all cases, only anti isomers were obtained. For both catalyst types decrease of efficiency and enantioselectivity was observed in case benzaldehydes substituted by electron-donating group (methoxy group) in para-position. For (*aR*,*R*)-**34a** two non-aromatic aldehydes were used–hexanal and *α*,*β*-unsaturated hex-2-enal. Great enantioselectivity 98% ee and good yield 83% has been received for the second one. For (*aS*,*R*)-**34a** observed that decreasing the amount from 10 mol% to 1 mol% with simultaneous elongation of the reaction time from 12 h to 24 h caused a slight decrease of both the yield and enantioselectivity (from 82%, 96% ee to 70%, 91% ee).

Zhao et al. have designed symmetrically and unsymmetrically substituted, axially chiral 2,2’- bipyridine *N*,*N’*-dioxides combined with central chirality introduced with *α*-amino acid residues: *C1*-symmetric **36** and *C2*-symmetric **35** (see Figure 7) [53]. Using 10 mol% of catalyst for allylation of *p*-nitrobenzaldehyde in CH_2_Cl_2_ at −78 °C (with the addition of DIPEA) rather mediocre results were obtained 41–66% yield, 27–40% ee. It was observed that the levorotary catalyst gave comparable or even slightly better results in shorter reaction times. Also, levorotary catalyst gave (*R*)-product, while dextrorotary catalyst gave (*S*)-product. Nine different polar and nonpolar solvents were tested in allylation using (−)- **35** as catalyst and CH_2_Cl_2_ (48%, 33% ee)_,_ next to MeCN (35%, 41% ee), seemed to be better solvents for the allylation reactions. But in MeCN, nearly 3 times longer reaction time was needed to achieve shown results (11 h versus 4 h). Catalyst (–)-**36b** showed similar enantioselectivity in the same test reaction, giving 66% yield and 35% ee, Table 6, entry 1). Both, *C2*-symmetric catalyst **35** and *C1*-symmetric catalyst **36b** have been evaluated in the allylation of other substituted benzaldehydes (see Table 6). The best results were obtained with 4-methylbenzaldehyde and 1,4-benzodioxane-6-carboxaldehyde–61–65% ee and 53–63% ee, respectively (Table 6, entries 6 and 8). By increasing the catalyst loading (for **35**, Table 6, entry 7), a bit higher yield was obtained, but a decrease in enantioselectivity was observed. The allylation of 4-methoxybenzaldehyde was also performed using (–)-**36b** and, despite that methoxy group is electron-donating (similar to methyl group), in this case, the obtained product was racemic. 

Kotora et al. synthesized a number of derivatives **34** (see Figure 7), with variously modified aromatic substituents at the 3’ position [54]. All these dioxide derivatives have been tested, as Lewis base catalysts, in allylation of aromatic aldehydes (0.5 mol%) in THF and CH_2_Cl_2_. Again, THF was a better reaction medium. As observed, the substitution of catalysts in the aryl group by EDG or EWG did not significantly change the catalytic activity of **34a**. Slightly better results were observed for all (*S*,*R*) isomers, except the results of cinnamaldehyde allylation, which were similar for both epimers of **34b**–**g**. Asymmetric induction was not observed in the allylation of 2-thiophenecarboxaldehyde. Gradual increase of catalyst loading showed, that both the yield and enantioselectivity increased up to 74%, 80% ee for 5 mol% of (*aR*,*R*)-**34a** and 90%, 88% ee for 5 mol% of (*aS*,*R*)-**34a**. The allylations of benzaldehyde, *p*-methoxybenzaldehyde and *p*- trifluoromethylbenzaldehyde using **34**, described in this report, were performed using 0.5 mol% of the catalyst [54]. Comparing them with the same reactions applying 1mol% of the catalyst [36] allows the conclusion that reducing the loading does not affect enantioselectivity. Only in case of *p*-trifluorobenzaldehyde the yield slightly decreased using (*S*,*R*)-enantiomer, while with use (*R*,*R*)-enantiomer, the yield apparently decreased, when half amount of catalyst has been used.

The predictable utility of the expected product as a chiral building block in the synthesis of natural products [54,55] was the reason for the application of **34a**–**g** in allylation of *E*-3-iodomethacrylaldehyde (Table 7). For this reaction, the influence of modification of the phenyl group in catalyst turned out to be significant. The advantage of (*aR*,*R*)-catalysts **34b**–**g** over (*aR*,*R*)-**34a** (with unsubstituted phenyl group in 3′ position) was especially noticeable in term of enantioselectivity (80–97% in comparison to 57%). When it comes to the yield, the highest result was observed for (*aR*,*R*)-**34e**, having 4-trifluoromethylphenyl group (Table 7, entry 5), while by using (*aR*,*R*)-**34b**, having 4-methoxyphenyl group (Table 7, entry 2), the lowest conversion was obtained. In all other cases, yields were comparable (approx. 70%). In the case of the (*aS*,*R*)-isomers, for all catalysts, including (*aS*,*R*)-**34a**, the obtained enantioselectivity was excellent (98–99% ee). For compounds with EWG or EDG substituent present at the phenyl group, an increase of yield was observed–from 49% to 66–89%. The best and the worst results coincided with those obtained for (*aR*,*R*)-enantiomers (Table 7, entries 5 and 2, respectively). The configuration of the obtained product was the same as the axial configuration in the catalyst.

Kotora and Lamaty presented bipyridine *N*,*N’*-dioxides **37**, with *C2*-symmetry (see Figure 7) [24,25]. Their synthesis was based on catalytic [2+2+2] cyclotrimerization either hepta-1,6-diyne or propargyl ether as starting material with various nitriles. A detailed synthetic pathway, including two different approaches, was described. Obtained compounds **37a**,**b** were examined in the allylation of benzaldehyde. Although the use of acetonitrile as solvent notably improved the yields, (93% with (*aR*,*S*,*S*)-**37b** and 96% with (*aR*,*S*,*S*)-**37a**), compared to results in THF, the enantioselectivity remained moderate (45% ee and 33% ee, respectively). On the other hand, using DCM rise in enantioselectivity (72% ee using (*aR*,*S*,*S*)-**37b** and 46% ee using (*aR*,*S*,*S*)-**37a**) with noticeably decreased the yield. In both cases, slightly better enantioselectivity was observed for (*aS*,*S*,*S*)-isomers of **37a**,**b**, but it was still rather moderate (51% ee and 40% ee respectively). 

**Scheme 9:**
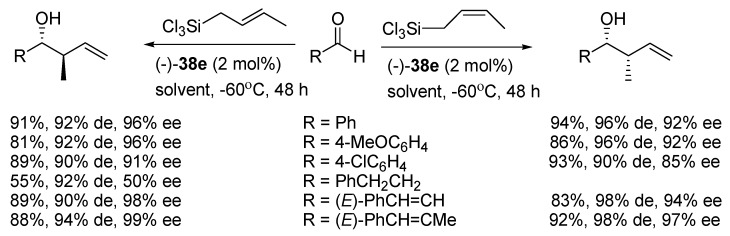
Asymmetric crotylation of aldehydes with (-)-**38e**.

A recent report by Rubtsov and Malkov et al. [56] presents the synthesis of atropoisomeric bipyridine *N*,*N*′-dioxides with terpene derived moieties **38** (see Figure 7). The combination of axial chirality and terpene-derived structures seemed to be a promising direction. Especially compound **38e** turned out to be an extremely efficient catalyst producing excellent enantio- and diastereoselectivities in the asymmetric crotylation over a whole range of aldehydes tested [14]. Catalyst (−)-**38e** proved particularly efficient with unsaturated aldehydes, though with aliphatic enantioselectivity dropped representing a common trend (see Scheme 9).

**Scheme 10:**
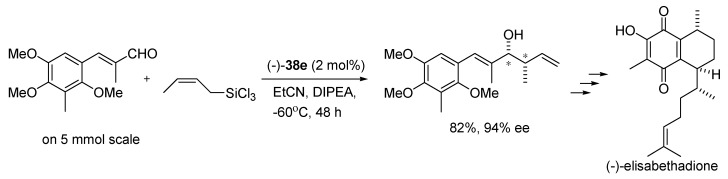
Crotylation with **38e** in the total synthesis of (-)-elisabethandione [14].

Applying (−)-**38e** in the same reaction conditions to a larger scale (5 mmol), provides excellent yield and enantioselectivity retained paving the way for the asymmetric synthesis of (−)-elisabethadione (see Scheme 10) [14].

**Scheme 11:**
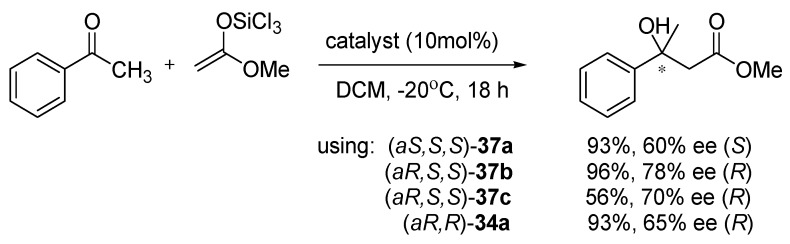
Catalytic aldol reaction of acetophenone with trichlorosilyl ketene acetal [24].

Compounds **37** were also applied in aldol reaction of acetophenone with trichlorosilyl ketene acetal (Scheme 11) [24]. Good to excellent yields were achieved using **37a**,**b** catalysts, independently of their configuration (87–96%). For **37a**, enantioselectivity was higher when (*aS*,*S*,*S*)-isomer was used and for **37b**,**c** with (*aR*,*S*,*S*)-isomer. The difference between the structure of catalysts is the presence of oxygen in the five-membered ring of compounds. The more selective were catalysts bearing the oxygen atom in the five-membered ring (**37b**,**c**). The best result received for (*aR*,*S*,*S*)-**37b** was 96% yield and 78% ee, which is better than obtained with the catalyst (*aR*,*R*)-**34a** in the same conditions. 

### 2.4. Helical-Chiral N-Oxides

**Figure 8:**
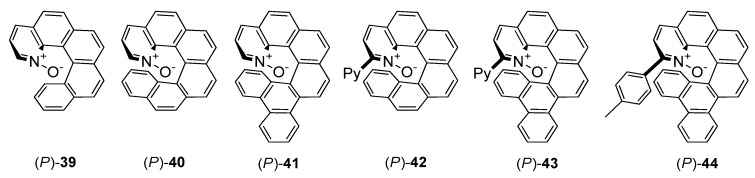
Helically chiral *N*-oxides.

A completely different group of *N*-oxide catalysts introduced Takenaka [21,22,23]. Seminal report from 2008, presented synthetic route to helical-chiral pyridine *N*-oxides **39**–**41** (see Figure 8) and results of their application in desymmetrization of meso-epoxides [21]. The catalytic reaction was performed for two epoxides possessing aromatic groups and two alkyl epoxides. In all cases the efficiency of the reaction was good (68–80%). Desymmetrization of aromatic meso-epoxides was characterized by higher enantioselectivity than for aliphatic ones (73–94% ee versus 22–65% ee). Introduction of two additional aromatic rings to **39**, creating branched structure **41**, allowed to obtain in all cases the highest ee values. For 1,5-cyclooctadiene oxide, the growth of enantioselectivity was the most visible–33% ee with **41**, in comparison to racemate obtained using **39**. Epoxide ring opening of several variously substituted cis-stilbene derivatives catalyzed by **41** was also examined. It could be concluded, that the presence of the electron-withdrawing group in *cis*-stilbene has no effect on enantioselectivity (2 examples, 92–94% ee) but the presence of electron-donating group slightly decreases the enantioselectivity (87% ee). 

**Scheme 12:**
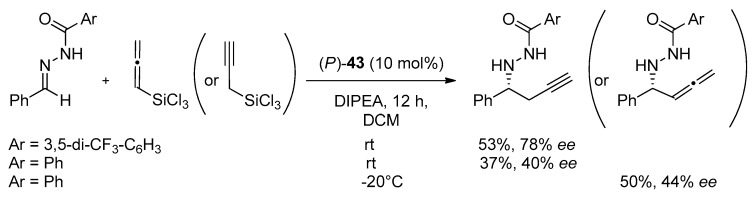
Propargylation and allenylation of *N*-acylhydrazones [23].

The same helical catalysts have been used in the propargylation of aldehydes with allenyltrichlorosilane [22]. Applying 10 mol% of catalyst in CH_2_Cl_2_ at −20 °C for 24 h, provided the products with good yield (80–90%) and moderate enantioselectivity (34–48% ee). Additionally, three new helically chiral *N*-oxides **42**–**44** were presented [22]. A slight modification of **40** and **41,** consisting of the insertion 2-pyridine group close to *N*-oxide bond (**42**, **43**, respectively) allowed to achieve complete conversion of aldehyde in 3 h but enhancement of the enantioselectivity (84% ee) was observed only for **43**. In comparison, using **44**, obtained by substitution of 4-methylphenyl group into **41** in position 2, the catalytic reaction did not proceed even at room temperature after 24 h. Compound **43** was applied to carry out the evaluation of the utility of different aromatic aldehydes as substrates [22]. For meta-substituted aldehydes, better enantioselectivity was obtained when substituents were electron-deficient. For ortho-substituted aldehydes, electron effects had no influence, but generally, the yield and enantioselectivity were slightly higher than for meta-substituted aldehydes, what is shown in Table 8. Catalyst **43** was also employed in propargylation of N-acylhydrazone (Scheme 12) with a satisfactory result–53% yield and 78% ee [23]. Results proved that helical chiral 2,2′-bipyridine N-monoxides that exhibited high activity towards the relatively unreactive allenyltrichlorosilane. The appropriate structural modification to the rings beneath the plane of the pyridine *N*-oxide can serve as a powerful means for tuning the catalyst enantioselectivity.

Pyridine *N*-oxides have been also used as a functional group in helically chiral organocatalysts supported on polymers. For example, pyridyl *N*-oxide substituted poly(methacrylate)s [57], poly(biphenylylacetylene)s [58] and D-glucose-linked biphenyl polymers [59] were reported. All these catalysts: monomers, polymers without N-O bond and polymers bearing *N*-oxide group, were examined in allylation of a few chosen aromatic aldehydes. Results in both, yields and asymmetric induction were rather low but, as a brief summary it should be emphasized, that presence of the *N*-O bond as well as the application of polymer-supported catalysts increase the enantioselectivity of the reaction.

### 2.5. N-Oxides Possessing Planar Chirality

**Figure 9:**
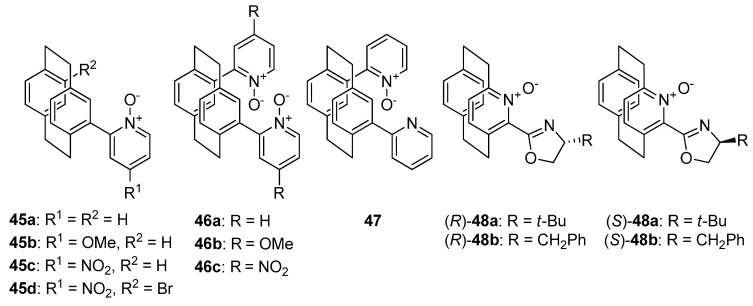
Planar chiral N-oxides.

Compounds bearing [2.2] paracyclophane moiety **45**–**48** (see Figure 9), which represents planar chiral N-oxides should also be briefly mentioned. Rowlands et al. presented a facile synthesis of the mentioned compounds in two steps from [2.2] paracyclophane, based on Fagnou’s direct arylation [60]. Then, obtained compounds were applied in the allylation of benzaldehyde with allyltrichlorosilane. For catalysts **45b** and **46b**, the presence of methoxy group in their structure caused decrease of the yield and enantioselectivity and also inversion of configuration from *R* to *S*, in comparison to corresponding unsubstituted structures **45a** and **46a**–from 65%, 38% ee to 52%, 36% ee and from 72%, 38% ee to 58%, 28% ee, respectively. Unsubstituted, mixed pyridine/pyridine *N*-oxide catalyst **47** was less effective (55% yield and 30% ee) compare to **46a** and inversion of product configuration was also observed, which might indicate that presence of *N*-oxide group plays a crucial role. It is also worth to compare the results obtained by Rowlands and coworkers with those obtained by Andrus and coworkers [61]. The latter group synthesized aza-paracyclophane *N*-oxide catalysts **48**. Among them (*S*)-**48a** turned out to be a brilliant catalyst for allylation of various aromatic and aliphatic aldehydes with allyltrichlorosilane, giving 87–95% yield and 87–96% ee [61]. It is puzzling whether a structure containing aza-paracyclophane *N*-oxide would be effective catalysts or the presence of an oxazoline group is essential. 

## 3. Chiral Heteroaromatic *N*-Oxides as Ligands for Metal Catalysis

**Figure 10:**
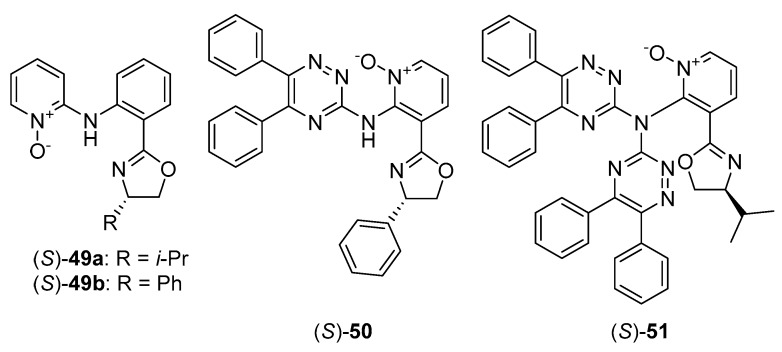
*N*-oxide ligands used in Cu-catalyzed nitroaldol reaction.

Over the past decade, relatively little attention has been paid to the use of chiral heteroaromatic *N*-oxides as ligands for metal complexes catalyzed reactions. Several mentions come from 2002–2003 and include (*S*)-**1**-CdI_2_ complex as a catalyst for conjugate addition of thiol to enone or enal, providing 70–78% ee [62], and (*R*)-**1**-Sc(OTf)_3_ complex as a catalyst for Michael addition of *β*-keto ester to methyl vinyl ketone or acrolein, giving almost quantitative yield with moderate enantioselectivity of 38–84% ee [48]. Chiral copper (II)-terpyridine mono-*N*-oxide and di-*N*-oxide complexes were used in asymmetric cyclopropanation of styrene [63]. Enantiomeric excess up to 83% and yield up to 97% were achieved. Currently, rather the applications of chiral alkyl amine *N*-oxides (mostly proline *N*-oxide derivatives) as ligands for metal complexes, in various types of asymmetric reactions, can be found in the literature [64,65,66]. The different direction was presented by Wolińska, who used chiral pyridine *N*-oxide derivatives possessing oxazoline moiety **49**–**51** (see Figure 10) for the asymmetric nitroaldol reaction, catalyzed by a copper complex. Catalysts **49**, used in Henry reaction of 3-nitrobenzaldehyde [67], gave high efficiency (80–88%) but unfortunately, the enantioselectivity was low (11–14% ee). Chiral 3-oxazoline pyridine *N*-oxides substituted by with 1,2,4-triazine ring (**50**, **51**) [68] were also examined in nitroaldol reaction of *m*-nitrobenzaldehyde but all attempts resulted in racemic nitroalcohol. An external base addition was tried and some improvement has been observed [69]. However, enantioselectivity grows (from racemate to 41% ee) only for bases with low pKa value. The reason for the low catalytic effectiveness may be the relatively large distance between the complexation site (N-O) and the stereogenic center.

## 4. Other Applications of Pyridine *N*-Oxides

Without any doubt, N-oxides are used in asymmetric organocatalysis, but also their applications in different branches of science are of great importance. They have significant synthetic value as intermediates in multi-step syntheses. They are widely used to various functionalization of *N*-heteroaromatic compounds-this mainly concerns the C-H bond in position 2. The examples of the application of *N*-oxides as synthetic intermediates in the industrial synthesis of some pharmaceuticals are also described, e.g., pranoprofen or omeprazole [70]. Compounds containing in their structure the 2-mercaptopyridine-*N*-oxide moiety have anti-cancer, bactericidal, and fungicidal activity [71,72]. *N*-oxides are also a crucial component in personal care products such as soaps, toothpaste, washing agents, shampoos and cosmetics [73]. Interesting properties of the *N-O* bond caused that N-oxides are used also in materials engineering. They consist of a wide group of polymer additives, e.g., crosslinkers, vulcanization accelerators, epoxy resin hardeners, UV absorbers or additives for stereospecific polymerization of polypropylene [74]. The most attention is focused on polymers with *N*-oxide groups e.g., hyperbranched polyimide *N*-oxide, which is used as photocatalyst [75]. Their greatest advantages, in comparison to photocatalysts based on inorganic compounds, are easy and cost-efficient synthesis and, particularly, the possibility of visible light absorption without the necessity of structural modifications. Another example of the photocatalyst is light crosslinked polymers, based on triazine *N*-oxide fragment. It has been shown that they are effective photocatalysts, causing degradation of methyl orange, an azo dye employed as a pH indicator [76]. Most dyes have a very stable structure, which makes their degradation especially difficult and uncontrolled entry of these compounds into water affects flora and fauna. In the case of water reservoirs where there is no flow of water, it might cause eutrophication. *N*-oxides are also used in coordination polymers, among which semiconductor luminescent materials with tunable luminescence are sought. This type of material can be applied in lighting and displays, as well as in-memory devices and sensors. As an example can be mentioned coordination polymers with symmetric and unsymmetrical ligands-4,4′- and 2,2′-bipyridine *N*,*N’*-dioxides and *N*-oxides [77]. Recent reports concern also pH-responsive polystyrene-b-poly(4-vinylpyridine-*N*-oxide) membranes [78] and the possibility of applying the coatings from a solution of cellulose-*N*-methylmorpholine-*N-oxide* to paper [79]. In the first case, at low pH the pores open (the solution flow increases), and at high pH, the pores close (the solution flow is reduced). The membrane is synthesized by oxidation of polystyrene-b-poly(4-vinylpyridine), which shows an inverse pH response and the presence of both forms in membrane opens up an attractive way for pH-based separations [78]. In other cases, depending on the composition of the coating and whether is it continuous or porous, it is possible to improve the tear strength, print quality as well as the adhesive or antibacterial properties of the paper. It also affects fire resistance, thermal and electrical conductivity, and the friction coefficient. Paper with a coating from a cellulose-*N*-oxide solution was characterized above all by higher hydrophobicity and smoothness of the surface as well as better tear resistance [79].

## 5. Conclusions

Chiral pyridine *N*-oxides and 2,2’-bipyridine *N*,*N’*-dioxides are very well suited as Lewis base organocatalysts. They are able to activate trichlorosilanes and catalyze a number of reactions such as allylation of aldehydes (furnishing homoallylic alcohols—main field), hydrosilylation, ring-opening of epoxides and aldol condensations. Their stereo-differentiating properties largely depend on the type of chirality represented by the molecules, but they can also be modified by the presence of substituents that slightly change the electron properties of the catalysts. However, the results reported in this paper clearly show that fine-tuning of the properties of the catalysts may not be as straightforward and simple as expected. The stereogenic axis in the catalyst appears to have the most pronounced effect on both, the product configuration and its optical purity. However, this effect also highly depends on the solvent used. Generally, N-oxides do not require the application of large amounts to have an acceptable effect compared to other organocatalysts. On average, loading ranges from 5–10 mol%, although sometimes 1 mol% is enough. Extensive optimization of the stereoselective features of the catalysts and reaction conditions has resulted in the high level of stereocontrol and yields in allylation of aromatic or unsaturated aldehydes, although good results have been obtained also for aliphatic, e.g., cyclic aldehydes. Allylation procedures employing N-oxide organocatalysts have found practical applications in the synthesis of natural products.

Although a variety of chiral pyridine *N*-oxide derivatives are able to perform reactions efficiently and with high enantioselectivity, they often need synthesis according to tedious procedures, sometimes involving a resolution step. Therefore, the developing of new structures, readily available and efficient as chiral organocatalysts for the reaction of trichlorosilyl compounds is still very active.

## Figures and Tables

**Table 1:**
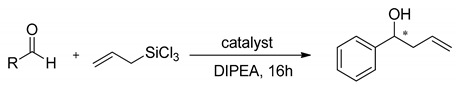
Asymmetric allylation of aldehydes with allyltrichlorosilane catalyzed by different *N*-oxides.

Entry	R	Catalyst, Yield [%], *ee* [%]					
		(*R*)-4a ^a^ [33]	(*R*)-4c ^a^ [34]	(*R*)-6h ^a^ [35]	(*S*)-8a ^b^ [36]	(*S*)-8g ^b^ [36]	(*S*,*S*)-12i ^c^ [37]
		1 mol%			0.05 mol%		20 mol%
1	Ph	88, 95 (*S*)	79, 95 (*S*)	85, 87 (*R*)	64, 88 (*R*)	-	87, 83 (*S*)
2	2-MeO-C_6_H_4_	-	-	86, 67 (*R*)	85, 86 (*R*)	-	95, 80 (*S*)
3	2-Cl-C_6_H_4_	-	-	-	58, 75 (*R*)	69, 82 (*R*)	-
4	2-CH_3_-C_6_H_4_	-	-	-	75, 86 (*R*)	-	-
5	2-F-C_6_H_4_	71, 95 (*S*)	76, 94 (*S*)	-	56, 80 (*R*)	76, 80 (*R*)	-
6	3-MeO-C_6_H_4_	-	80, 97 (*S*)	90, 83 (*R*)	80, 94 (*R*)	-	-
7	3-Cl-C_6_H_4_	74, 97 (*S*)	74, 96 (*S*)	80, 82 (*R*)	47, 78 (*R*)	70, 82 (*R*)	-
8	3-CH_3_-C_6_H_4_	-	-	-	81, 94 (*R*)	-	-
9	3-Br-C_6_H_4_	-	-	-	48, 80 (*R*)	62, 80 (*R*)	-
10	3-NO_2_-C_6_H_4_	-	80, 93 (*S*)	97, 96 (*R*)	-	-	-
11	4-MeO-C_6_H_4_	75, 99 (*S*)	83, 99 (*S*)	88, 94 (*R*)	90, 96 (*R*)	71, 94 ^d^ (*R*)	84, 87 (*S*)
12	4-Cl-C_6_H_4_	77, 97 (*S*)	77, 96 (*S*)	85, 76 (*R*)	62, 86 (*R*)	-	98, 76 (*S*)
13	4-CH_3_-C_6_H_4_	74, 95 (*S*)	75, 95 (*S*)	90, 76 (*R*)	76, 96 (*R*)	-	-
14	4-F-C_6_H_4_	76, 97 (*S*)	-	88, 73 (*R*)	-	-	-
15	4-NO_2_-C_6_H_4_	-	83, 88 (*S*)	95, 86 (*R*)	-	-	47, 25 (*S*)
16	2,4-di-MeO-C_6_H_3_	-	-	-	85, 96 (*R*)	-	-
17	3,4-di-MeO-C_6_H_3_	-	87, 96 (*S*)	-	-	-	81, 94 (*S*)
18	2,6-di-Cl-C_6_H_3_	87, 97 (*R*)	82, 97 (*R*)	-	-	-	-
19	2-furyl	-	-	-	52, 56 (*R*)	-	52, 93 (*R*)
20	2-thiophenyl	83, 97 (*S*)	80, 98 (*S*)	-	81, 96 ^e^ (*R*)	-	-
21	2-thienyl	-	-	-	-	-	71, 92 (*R*)
22	3-thienyl	-	-	-	-	-	67, 89 (*S*)
23	1-naphthyl	82, 96 (*S*)	85, 97 (*S*)	-	79, 94 (*R*)	-	-
24	2-naphthyl	-	88, 93 (*S*)	87, 76 (*R*)	-	-	-
25	9-anthryl	-	-	-	-	-	62, 92 (*S*)
26	*N*-acetyl-3-indolyl	-	-	-	72, 98 ^f^ (*R*)	-	-
27	piperonyl	-	-	-	77, 96 (*R*)	-	58, 81 (*S*)
28	*E*-PhCH = CH	90, 91 (*S*)	92, 93 (*S*)	-	84, 92 (*R*)	-	37, 71 (*S*)
29	*E*-PhCH = C(CH_3_)	-	-	-	79, 96 (*R*)	-	-
30	*E*-PhCH = C(Br)	-	-	-	96, 98 ^f^ (*R*)	-	-
31	PhCH_2_-CH_2_	73, 92 (*R*)	64, 92 (*R*)	-	-	-	-
32	*c*-C_6_H_11_	53, 97 (*S*)	50, 92 (*S*)	NR ^g^, 40 (*R*)	-	-	64, 33 (*S*)

^a^ Reactions were performed in DCM at −80 °C. ^b^ Reactions were performed in a mixture of solvents—1.0 M in MeCN:THF, 3:1 at −40 °C. ^c^ Reactions were performed in CHCl_3_: Cl_2_(CH)_2_Cl_2_, 1:1 at −78 °C for 24 h with the addition of Bu_4_N^+^I^−^. ^d^ 2.5 h. ^e^ 0.5 mol%. ^f^ 0.1 mol%, 71 h. ^g^ Not reported in [35].

**Table 2:**
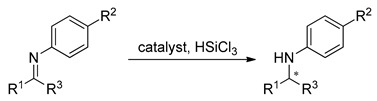
Enantioselective hydrosilylation of various ketoimines catalyzed by (*R*)-**6i** and **7**.

Entry	R^1^, R^2^, R^3^	Catalyst, Yield [%], ee [%]	
		(*R*)-6i ^a^, 1 mol% [26]	7 ^b^, 20 mol% [27]
1	Ph, H, Me	97, 83 (*S*)	95, 93 (*S*)
2	4-F-C_6_H_4_, H, Me	98, 68 (*S*)	98, 95 (*S*)
3	4-Cl-C_6_H_4_, H, Me	97, 71 (*S*)	97, 94 (*S*)
4	4-Br-C_6_H_4_, H, Me	96, 80 (*S*)	96, 93 (*S*)
5	4-Br-C_6_H_4_, MeO, Me	-	98, 76 (*S*)
6	4-CF3-C_6_H_4_, H, Me	-	95, 84 (*S*)
7	4-NO2-C_6_H_4_, H, Me	96, 67 (*R*)	95, 78 (*S*)
8	4-MeO-C_6_H_4_, H, Me	95, 85 (*S*)	94, 77 (*S*)
9	Ph, MeO, Me	97, 75 (*S*)	97, 96 (*S*)
10	Ph, EtO, Me	97, 82 (*S*)	95, 94 (*S*)
11	Ph, Me, Me	99, 84 (*R*)	95, 93 (*S*)
12	Ph, Et, Me	97, 71 (*S*)	95, 89 (*S*)
13	Ph, Br, Me	98, 71 (*R*)	98, 75 (*S*)
14	Ph, H, Et	95, 81 (*S*)	95, 94 (*S*)

^a^ Reactions were performed in CH_2_Cl_2_ at 0 °C, 16 h. ^b^ Reactions were performed in CHCl_3_ at rt, 4 h.

**Table 3:**
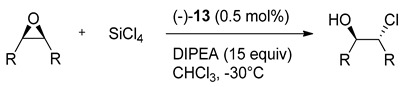
Desymmetrization of meso-epoxides with SiCl_4_ catalyzed by **13 [18]**.

Entry	R	Time [min]	Yield [%]	ee [%]	Configuration
1	Ph	120	94	93	1*R*,2*R*
2	3-MeO-Ph	90	96	89	1*R*,2*R*
3	4-Me-Ph	105	92	89	1*S*,2*S*
4	4-F-Ph	180	97	78	1*S*,2*S*
5	-(CH_2_)_3_-	45	67	22	-
6	-(CH_2_)_4_-	60	65	42	1*R*,2*R*
7	-(CH_2_)_6_-	90	84	69	1*R*,2*R*

**Table 4:**
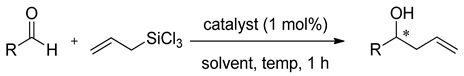
Asymmetric allylation of aldehydes by **32** [50] and **34a [31]**.

Entry	R	Solvent	T [°C]	Catalyst, Yield [%], ee [%]			
				(*aR*,*R*,*R*)-32	(*aS*,*R*,*R*)-32	(*aR*,*R*)-34a	(*aS*,*R*)-34a
1	Ph	MeCN	–40	100, 48 (*S*)	100, 46 (*R*)	100, 53 (*S*)	100, 44 (*R*)
2	Ph	PhCl	–40	0, 0	100, 62 (*S*)	100, 93 (*R*)	100, 94 (*S*)
3	Ph	THF	–78	-	-	100, 93 (*R*)	98, 96 (*S*)
4	4-MeO-C_6_H_4_	MeCN	–40	100, 60 (*S*)	100, 0	-	-
5	4-MeO-C_6_H_4_	PhCl	–40	0, 0	47, 56 (*S*)	93, 55 (*R*)	-
6	4-MeO-C_6_H_4_	THF	–78	-	-	100, 86 (*R*)	90, 91 (*S*)
7	4-Me-C_6_H_4_	THF	–78	-	-	99, 85 (*R*)	99, 94 (*S*)
8	4-F-C_6_H_4_	THF	–78	-	-	81, 84 (*R*)	99, 95 (*S*)
9	4-CF_3_-C_6_H_4_	THF	–78	-	-	76, 95 (*R*)	91, 87 (*S*)
10	4-CF_3_-C_6_H_4_	MeCN	–40	82, 15 (*R*)	100, 16 (*R*)	-	-
11	4-CF_3_-C_6_H_4_	PhCl	–40	0, 0	40, 75 (*S*)	76, 67 (*R*)	-
12	4-CN-C_6_H_4_	THF	–78	-	-	55, 83 (*R*)	93, 88 (*S*)
13	4-Cl-C_6_H_4_	THF	–78	-	-	89, 89 (*R*)	91, 93 (*S*)
14	3-Cl-C_6_H_4_	THF	–78	-	-	75, 87 (*R*)	78, 92 (*S*)
15	2-Cl-C_6_H_4_	THF	–78	-	-	45, 46 (*R*)	42, 14 (*S*)

**Table 5:**
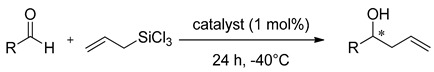
Asymmetric allylation of aliphatic aldehydes by **32** [30] and **34a [31]**.

Entry	R	Catalyst	Solvent	Yield [%]	ee [%]
1	*c*-C_6_H_11_	(*aR*,*R*,*R*)-**32**	MeCN	85	13 (*S*)
2			CH_2_Cl_2_	40	19 (*S*)
3			CHCl_3_	36	10 (*S*)
4			acetone	34	13 (*S*)
5		(*aS*,*R*,*R*)-**32**	MeCN	67	39 (*R*)
6			CH_2_Cl_2_	79	65 (*R*)
7			CHCl_3_	52	68 (*R*)
8			acetone	77	60 (*R*)
9	*n*-C_5_H_11_	(*aS*,*R*,*R*)-**32**	CHCl_3_	84	56 (*S*)
10	*n*-C_7_H_15_			79	64 (*S*)
11	*c*-C_5_H_9_			91	68 (*R*)
12	*c*-Pr			90	42 (*R*)
13	*t*-Bu			10	22 (*S*)
14	*n*-C_7_H_15_	(*aR*,*R*)-**34a**	CHCl_3_	90	60 (*S*)
15	*c*-C_5_H_9_			95	67 (*R*)
16	*n*-C_7_H_15_	(*aS*,*R*)-**34a**	CHCl_3_	88	43 (*S*)
17	*c*-C_5_H_9_			94	38 (*R*)

**Table 6:** Asymmetric allylation of benzaldehydes with allyltrichlorosilane catalyzed by **35** and (–)-**36b [53]**.

Entry	Aldehyde	Catalyst (10 mol%), Yield [%], ee [%]		
		(–)-35 ^a^	(+)-35 ^a^	(–)-36b ^b^
1	4-NO_2_-C_6_H_4_CHO	48, 33 (*R*)	41, 27 (*S*)	66, 35 (*R*)
2	4-Br-C_6_H_4_CHO	-	-	67, 42 (*R*)
3	4-Cl-C_6_H_4_CHO	31, 49 (*R*)	67, 51 (*S*)	54, 49 (*R*)
4	3-Cl-C_6_H_4_CHO	46, 43 (*S*)	32, 39 (*R*)	58, 45 (*S*)
5	4-MeO-C_6_H_4_CHO	-	-	58, 3 (*R*)
6	4-Me-C_6_H_4_CHO	23, 61 (*S*)	30, 65 (*R*)	65, 65 (*S*)
7		46, 53 (S) ^c^	36, 53 (*R*) ^c^	-
8	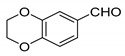	14, 53 (ND) ^d^	44, 59 (ND) ^d^	61, 63 (ND) ^d^
9		40, 51 (ND) ^c,d^	59, 61 (ND) ^c,d^	-

^a^ Reaction was carried out with 10 mol% of catalysts in CH_2_Cl_2_ at −78 °C for 5–10 h. ^b^ Reaction was carried out with 10 mol% of catalysts in CH_2_Cl_2_ at −78 °C for 8–12 h. ^c^ 20 mol% catalyst was used. ^d^ Not determined.

**Table 7:**
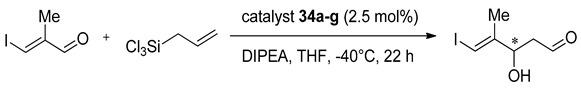
Allylation of *E*-3-iodomethacrylaldehyde catalyzed by **34a**–**g** [54].

Entry	Catalyst	Yield [%], ee [%]	Catalyst	Yield [%], ee [%]
1	(*aR*,*R*)-**34a**	71, 57 (*R*)	(*aS*,*R*)-3**4a** ^a^	49, 98 (*S*)
2	(*aR*,*R*)-**34b**	58, 85 (*R*)	(*aS*,*R*)-**34b**	66, 98 (*S*)
3	(*aR*,*R*)-**34c**	72, 96 (*R*)	(*aS*,*R*)-**34c**	77, 98 (*S*)
4	(*aR*,*R*)-**34d**	71, 97 (*R*)	(*aS*,*R*)-**34d**	79, 98 (*S*)
5	(*aR*,*R*)-**34e**	85, 94 (*R*)	(*aS*,*R*)-**34e**	89, 99 (*S*)
6	(*aR*,*R*)-**34f**	70, 80 (*R*)	(*aS*,*R*)-**34f**	76, 98 (*S*)
7	(*aR*,*R*)-**34g**	74, 88 (*R*)	(*aS*,*R*)-**34g**	-

^a^ Reaction time: 45 h.

**Table 8:**
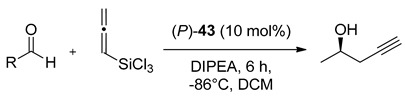
Asymmetric propargylation of aldehydes catalyzed by (*P*)-**43 [23]**.

Entry	R	Yield [%]	ee [%]	Entry	R	Yield [%]	ee [%]
1	2-Br-C_6_H_4_	93	96	9	2-NO_2_-C_6_H_4_	87	96
2	4-Br-C_6_H_4_	95	92	10	4-NO_2_-C_6_H_4_	55	92
3	2-Cl-C_6_H_4_	97	96	11	2-MeO-C_6_H_4_	78	94
4	4-Cl-C_6_H_4_	90	92	12	4-MeO-C_6_H_4_	80	74
5	2-F-C_6_H_4_	98	92	13	2-Me-C_6_H_4_	90	86
6	4-F-C_6_H_4_	93	88	14	4-Me-C_6_H_4_	85	82
7	2-CF_3_-C_6_H_4_	95	94				
8	4-CF_3_-C_6_H_4_	80	90				
